# Electrophysical Properties and Structure of Natural Disordered *sp*^2^ Carbon

**DOI:** 10.3390/nano12213797

**Published:** 2022-10-27

**Authors:** Yevgeny A. Golubev, Igor V. Antonets

**Affiliations:** 1 Institute of Geology of Komi SC, Russian Academy of Sciences, 167982 Syktyvkar, Russia; 2 Department of Radiophysics, Syktyvkar State University, 167000 Syktyvkar, Russia

**Keywords:** natural disordered *sp*^2^ carbon, molecular and super-molecular structure, electrical and thermal conductivity, applications

## Abstract

The progress in the practical use of glassy carbon materials has led to a considerable interest in understanding the nature of their physical properties. The electrophysical properties are among the most demanded properties. However, obtaining such materials is associated with expensive and dirty processes. In nature, in the course of geological processes, disordered *sp*^2^ carbon substances were formed, the structure of which is in many respects similar to the structure of glassy carbon and black carbon, and the electrical properties are distinguished by a high-energy storage potential and a high efficiency of shielding electromagnetic radiation. Given the huge natural reserves of such carbon (for example, in the shungite rocks of Karelia) and the relative cheapness and ease of producing materials from it, the study of potential technological applications and the disclosure of some unique electrophysical properties are of considerable interest. In this paper, we present an overview of recent studies on the structure, electrophysical properties, and technological applications of natural disordered *sp*^2^ carbon with the addition of novel authors’ results.

## 1. Introduction

In recent years, the number of studies reporting the successful use of disordered *sp*^2^ carbon (D*sp*^2^C) materials in various technological processes is growing. A recent review article about the use of synthetic glassy carbon in advanced technological applications [1] clearly shows rapid technological progress in this field. At the same time, the production of materials with a glassy carbon structure is associated with several environmentally dirty processes. In the earth’s crust, “ready-made” D*sp*^2^C is quite common, and its use in technology will be cheaper and more environmentally friendly than its synthetic structural counterparts. In addition, the natural origin provides a variety of precursors and physicochemical characteristics of formation processes in different geological settings, which allows reducing the cost of laboratory production of certain types of carbon materials to study the effect of changes in various structural and chemical parameters in the experiment. One of the most demanded properties of D*sp*^2^C is its electrical conductivity, which, in combination with thermal and chemical stability, gives good prospects in the industry. This paper discusses the origin, structure, and electrical properties of naturally occurring disordered *sp*^2^ carbon and basic areas for application of its electrical properties.

## 2. Formation of Disordered *sp*^2^ Carbon in Nature: Thermodynamic Conditions, Carbon Sources, Processes

**Figure 1:**
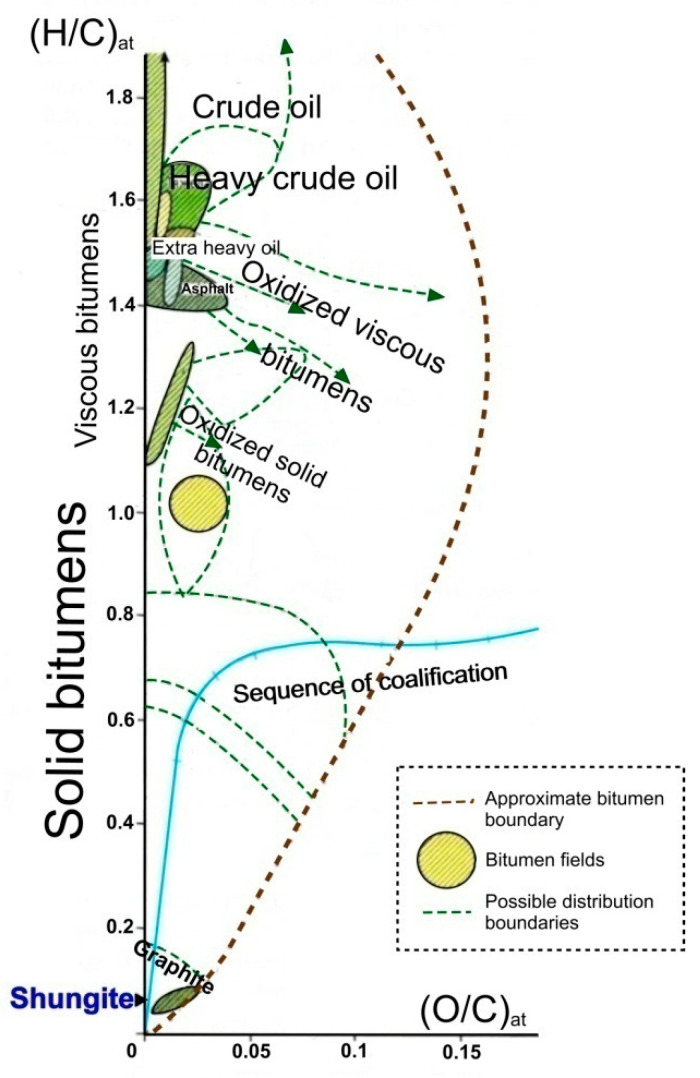
Van Crevelen diagram of carbonization (dehydrogenation) showing the alteration of H/C atomic ratios versus O/C. D*sp*^2^C (represented by shungite) is limited to a narrow region close to the end member of pure carbon (adapted from [2]).

Hydrocarbon compounds (primarily petroleum and bitumen) under the physicochemical conditions of the earth’s crust are transformed in the direction of carbonization (or dehydrogenation), with the elimination of most non-carbon components (Figure 1) [2], and in the direction of graphitization as a structural rearrangement of the aromatic skeleton formed during carbonization into a stable-layered graphite structure (*sp*^2^-hybridized) [3,4,5]. The process of graphitization of hydrocarbon compounds is irreversible, but it is not always terminated with the formation of graphite, both in terms of structure ordering and in terms of removal of hydrogen, oxygen, nitrogen, and sulfur heterocompounds. Condensation of carbonaceous matter can occur at the stage of formation of graphite-like nanostructures of various degrees of order and sizes [6] and can also end with the formation of structures such as multilayer ribbons and fullerenes [7,8]. In the sequence of carbonization (dehydrogenation) of hydrocarbon compounds, according to their physicochemical properties, a class of substances of the highest degree of transformation (carbonization) was distinguished, which was characterized by the so-called pre-graphite structural stage [9,10,11]. Such substances are distinguished by an almost pure carbon composition (usually over 95%) and the absence of solubility in organic solvents (for example, chloroform), and their distinctive physical property is electrical conductivity. According to the indicated properties, these substances are similar to graphite, while their critical difference with graphite is the absence of a bulk crystal structure (three-dimensional *hkl* reflections on X-ray and electron diffraction patterns). There is only two-dimensional graphite-like ordering as the proximity of interplanar distances to the 002 graphite plane. D*sp*^2^C is porous, and accordingly has a lower density (1.5–2.2) and a larger specific surface area than graphite.

**Figure 2:**
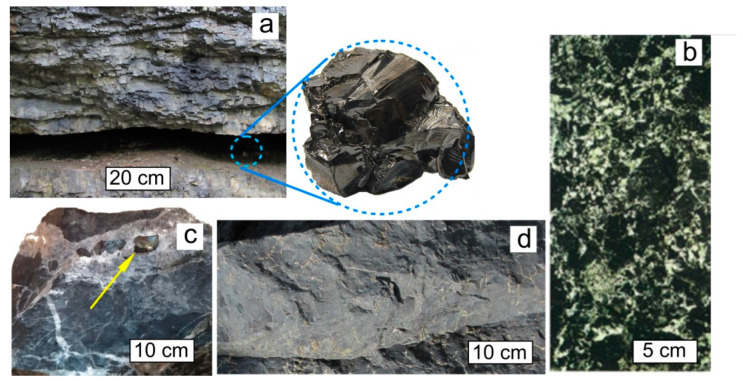
Typical occurrences of natural D*sp*^2^C: shungite carbon vein (Shunga occurrence, Karelia), with a typical sample in the inset (**a**). Cluster form of inclusions of D*sp*^2^C in the rock, Zazhogino occurrence, Karelia (**b**). D*sp*^2^C isolations as grains (indicated by yellow arrow) in a calcite vein, occurrence Rucheynoye, Kozhim River, Subpolar Urals, Russia (**c**). Rock (quartzite) impregnated with D*sp*^2^C, Maksovo occurrence, Karelia (**d**).

D*sp*^2^C is often present in oil and gas basins and in ore deposits of hydrothermal origin [11,12,13]. The geological conditions for the formation of such carbon in petroleum and gas source formations are deep catagenesis, metamorphism, intrusive magmatism, and post-magmatic hydrothermal activity [14,15,16]. The occurrences are known in rocks of all ages, including the most ancient Precambrian paleobasins, more than 2 billion years old. The scale of occurrences of D*sp*^2^C in the Earth’s crust varies over a very wide range. D*sp*^2^C both fills micro-sized pores in rocks and forms multi-meter reservoir deposits and large veins tens and hundreds of meters long (Figure 2). The occurrences of D*sp*^2^C have been found on all continents. The most fully characterized in the literature is the non-crystalline carbon of shungite occurrences in Karelia (Lake Onega region, Russia) [17,18]. Interest in shungite carbon increased after the first discovery of fullerenes of geological origin in it [19]. In addition to the shungite rocks of Karelia, significant occurrences with well-characterized structure and properties of D*sp*^2^C (referred to in the geological and mineralogical literature as anthraxolites and pyrobitumen) are located in Bakyrchik (Kyzyl fault zone, Kazakhstan) [20,21,22], Mitov (Bohemian massif, Czech Republic) [23,24], Novaya Zemlya island (Russia) [25], Sichuan province (China) [26], the Sudbury Astroblem (Canada) [27,28,29], the Franceville Sedimentary Basin (Gabon) [30,31], the Michigan Formation (USA) [32,33], and many others.

The formation of D*sp*^2^C is associated with volcanic or high-temperature hydrothermal metamorphism of hydrocarbons in or near petroleum deposits. D*sp*^2^C is a derivative of petroleum or substances similar to petroleum (for example, viscous bitumen) formed during the natural pyrolysis of kerogens under severe thermal and/or baric conditions, since their metamorphism occurs at depth. Additionally, the thermal impact of volcanic intrusions or their supply channels on bitumen occurrences in the surface layers of the Earth’s crust is possible. The precursors of D*sp*^2^C (petroleum and viscous bitumen) mainly originate from lacustrine algae and marine microorganisms, for example, during local contact heating of sapropelic algae massifs by volcanic intrusions or hydrotherms. The organic matter of terrestrial plants, as a rule, is transformed to coal, which is not the object of consideration in our work. Graphitizable carbon is characterized by precursors enriched in hydrogen, while non-graphitizable carbon has precursors with a more complex composition, enriched primarily in oxygen and sulfur [34].

Physicochemical conditions for the formation of D*sp*^2^C in nature are estimated from geological records. The lowest temperature stage of formation is the greenschist facies with a temperature range of 250–550 °C at a pressure of 1.5 to 4 kbar. In the hydrothermal process, the transformation occurs at a temperature of about 360 °C and a little higher. During volcanic contact metamorphism, the temperature of impact on hydrocarbon matter is the highest in the Earth’s crust. In the zone of contact between rocks and magma, an area of rock heating is formed, where the temperature reaches 500–900 °C. The maximum range of such metamorphism is 2–5 km. In rare cases, the formation of disordered *sp*^2^ carbon is accompanied by a local increase in pressure in the zones of tectonic faults [11]. This is conditioned by the fact that pressure accelerates graphitization, and under such conditions, the formation of well-crystallized graphite is more likely than D*sp*^2^C [35,36,37].

## 3. Structural Characteristics and Models of Natural Disordered *sp*^2^ Carbon

The object of our consideration is D*sp*^2^C, which makes its structural characteristics ambiguous. It does not graphitize during high-temperature (up to 2900 °C) treatment [38] under normal pressure conditions. The development of physicochemical methods allowed determining some structural features and chemical composition, but electron and X-ray amorphism still does not allow to unambiguously describe its structure. For example, at different times, the carbon of shungites was defined by researchers as weakly ordered graphite [39], amorphous carbon [4], natural metastable non-graphitic carbon [40], natural glassy carbon [41], and fullerene-like carbon in the group of non-graphitic natural carbonaceous substances [42,43]. In this section, the main models of the structure of natural D*sp*^2^C are considered, primarily using shungite carbon as an example.

### 3.1. Molecular Structure

**Figure 3:**
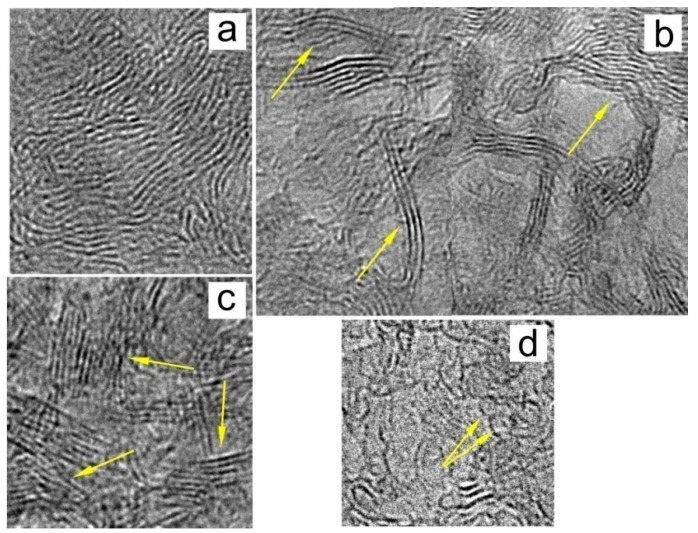
Basic structures of D*sp*^2^C: (**a**) typical entangled structure, (**b**) ribbon structure, (**c**) stacks of graphene layers, and (**d**) fullerene-like single-layer and multilayer structures.

The basis of the molecular structure of natural D*sp*^2^C is a graphene grid. The size and shape of these grids vary depending on the type of supramolecular structure to which the grid belongs. The following main supramolecular structures were found in natural D*sp*^2^C: turbostratic stacks of graphene layers (grids), multilayer ribbons, fully enclosed multilayer globules, and partially closed multilayer cups (Figure 3).

**Figure 4:**
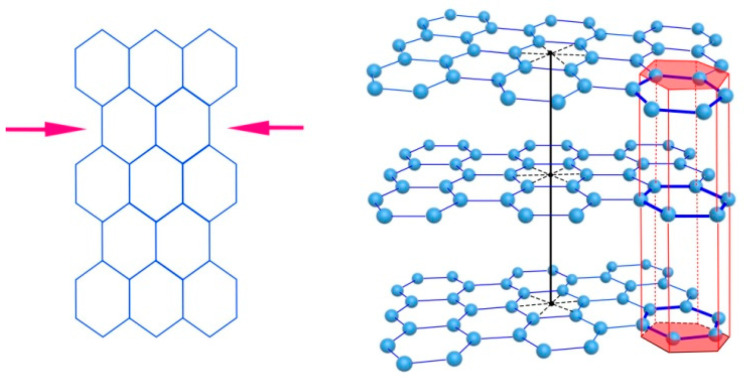
Deformation of a graphene layer (**left**) and displacement of flat graphene layers relative to each other in the stack (**right**).

In stacks, grids are predominantly 1–7 nm in size, and interlayer (interplanar) distances range from 0.341 to 0.360 nm (according to Kovalevsky [42,44] and Aleshina and Fofanov [45]). This exceeds the interplanar distances characteristic of graphite (0.335 nm). The shape of grids 1–2 nm in size is predominantly flat and even; however, with a further increase in size, the number of curved and crumpled grids increases. There are defects in the subtraction and insertion of grids within the graphene packs. It is likely that such local distortions of various nature result in the deformation of the internal structure of the graphene grid. For example, according to Kovalevsky, the carbon layer in the graphene grid is deformed by the type of compression and tension in different directions, which leads to a decrease in the hexagonal symmetry of the graphene layer to trigonal [43] (Figure 4).

In the stacks, the layers are turbostratically arranged. They are slightly deployed and (possibly) displaced relative to each other (Figure 4). As the reason for such an arrangement of layers, one can indicate the effect of edge functional groups. During the formation of carbon from the initial hydrocarbon substance, the structure of the graphene network condensed due to the destruction of the oxygen and hydrogen components. However, these functional groups (primarily oxygen groups) terminated the edges of graphene grids and prevented their further growth. Additionally, the van der Waals interaction of functional groups located in adjacent layers of packs “unfolded” the graphene layers relative to each other, preventing them from forming three-dimensionally ordered graphite nanocrystals. Such a pack can also be represented as an element of the supramolecular structure of D*sp*^2^C, since it consists of graphene molecules linked by non-covalent bonds.

The calculation from X-ray diffraction curves allowed determining that the grids were either flat (in this case, they consisted of 280–300 atoms) or curved (consisted of 180–200 atoms), and formed packets of five to six layers, within which the grids are chaotically displaced and misoriented relative to each other [45]. Some nodes, both in flat and curved grids, are vacant (their share of the total number is 5–15%). The deformation of the graphene layer can also be associated with the presence of pentagonal or heptagonal ring structures in it. If the graphene layer simultaneously contains pentagonal and heptagonal ring structures, then the layer will be bent in different directions, i.e., corrugated. Similar structures have been found in addition to shungites in the anthraxolites from Novaya Zemlya, Kazakhstan, Mitov, and others.

**Figure 5:**
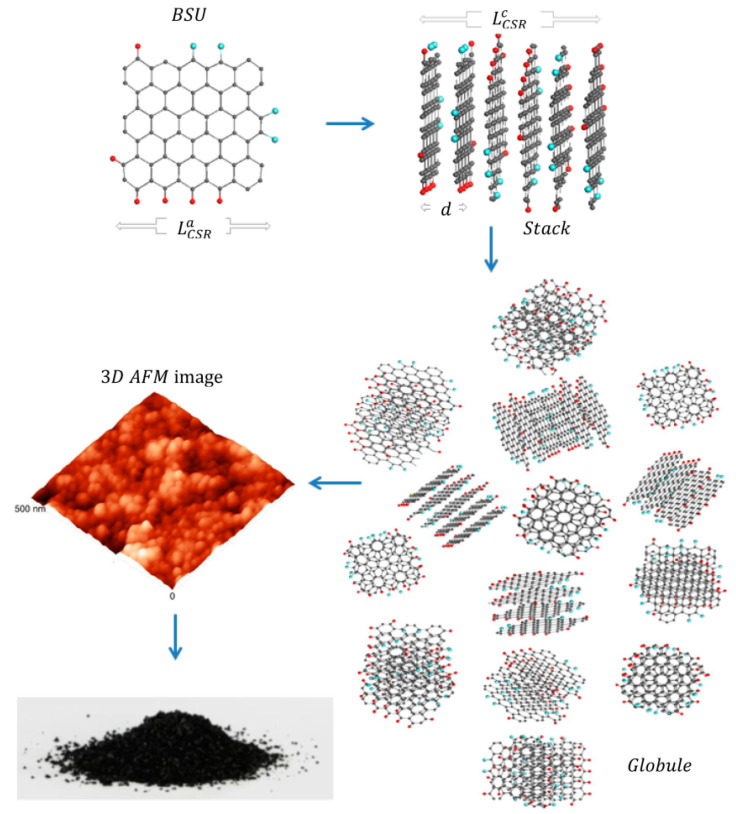
Scheme of the formation of a super-molecular globular structure of natural D*sp*^2^C (from [52]) through the formation of a stack of graphene layers as basic structural units (BSU) with subsequent aggregation of stacks into a rounded particle (globule). The globules are shown in an atomic force microscope (AFM) image. Along the edges of graphene molecules are heteroatoms (hydrogen (red balls) and oxygen (blue balls)).

In a series of works [46,47], shungite carbon is presented as a fractal multilevel structure. The authors consider fragments of reduced graphene oxide (RGO) with linear dimensions of ~1 nm as its main elements. Here, the structure of shungite carbon is based on nanosized RGOs, which, like their synthetic analogs [47,48], are formed as a result of a sequence of chemical transformations associated with the removal of oxygen-containing groups from the basal planes of graphene grids (Figure 5). The initial product for RGO formation was nanosized carbon fragments (lamellae). Simple oxygen-containing groups from the surrounding aqueous fluid acted as lamella oxidizers. The restoration of the lamellae took place under hydrothermal conditions for a long geological time, which ensured the occurrence of chemical reactions to their final stages. The difference between this graphene grid model and the work of Aleshina, Fofanova, and Kovalevsky lies primarily in the fact that in the model of Sheka, the graphene grid is assumed to be flat with a necklace of heteroatoms along the edges. The second structural level consists of five- to six-layer stacks of the indicated RGO fragment. Globules composed of these stacks, with linear dimensions of 3–6 nm, form the third structural level, and aggregates of globules, detected by scanning probe microscopy [49,50,51] and having sizes from 20 to 100 nm, form the final structural level.

**Figure 6:**
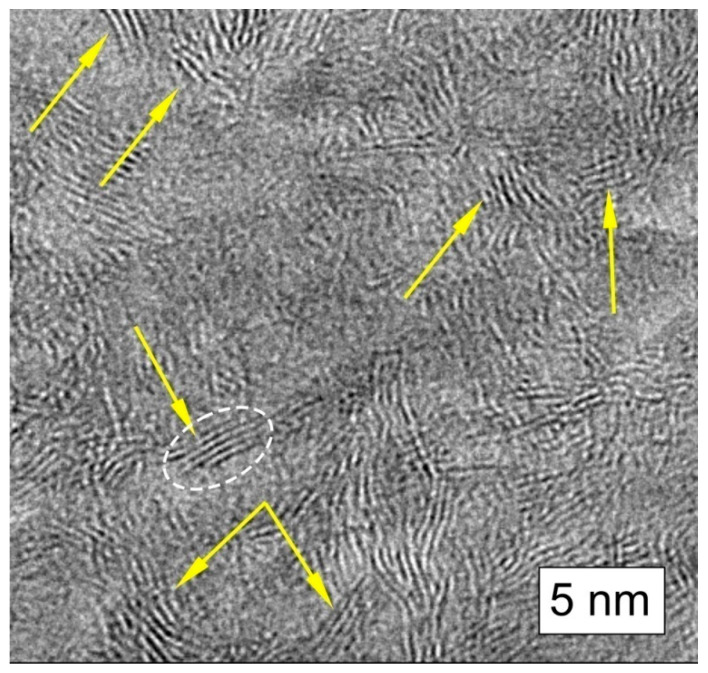
Chaotically oriented stacks of graphene layers in shungite carbon (indicated by yellow arrow).

Such models are confirmed, in particular, in the data of high-resolution transmission electron microscopy (HRTEM) [42,48,53,54,55]. Modern high-resolution images of the structure of natural D*sp*^2^C show a significant proportion of rectilinear bands up to several nanometers long. These bands are projections of the carbon atomic planes oriented almost parallel to the electron beam, which were referred in [48] to the basic nanosized RGO. Figure 6 shows that these planes are grouped into stacks. The distances between planes in stacks are 0.34–0.38 nm. Figure 6 clearly shows the grouping of atomic planes into stacks of 4–7 layers, which in the early works of Kovalevsky were interpreted as stacks of graphene layers [42]. Generally, in the shungite, the carbon layers as a rule are combined into stacks of 5–14 layers. Obviously, these stacks are misoriented relative to each other in the image plane. Due to the difference in the orientation of the stacks relative to the direction of the electron beam, some of them may not be visible under these measurement conditions. This hinders the estimation of the distribution density of such fragments; however, it seems to us that it is these piles that are the dominant structural element of D*sp*^2^C in the size range of 1–10 nm.

The Raman spectroscopy also specifies a similar structure of nanosized stacks as another very informative method to study the structure of D*sp*^2^C. In the 21st century, Raman scattering has rapidly become the dominant method for testing carbon solids based on graphene. Two approaches have been developed to understand Raman scattering in such structures. The solid-state approach is formed based on phonon scattering in both perfect and defective crystals, while the molecular approach is based on the analysis of vibrations of free molecules (for a review, see [56]). The similarity of the spectra for defective and highly disordered graphite crystals, represented by the D, G, and 2D key bands, allowed using and improving the solid-state approach to characterize the *sp*^2^ carbon structure, including the nanoscale one, using the position, width, and intensity of these bands. The solid-state approach was also applied to D*sp*^2^C. But, due to the structure disorderness and the small size of the graphene layers packs the correctness of this approach is weak in comparison the molecular approach to the Raman spectra of natural D*sp*^2^C [57,58]. In general, despite the difference in theoretical foundations, the key result of the interpretation of Raman spectra in the framework of both approaches is the assessment of the structure of natural D*sp*^2^C as a set of stacks of graphene layers with sizes of a few nanometers [55,57,58]. It should be noted that a reliable estimate of the packet sizes from Raman spectra at the current stage causes great difficulties for the objects of our study [57,58].

In [5,52], as a result of a comprehensive study of a wide range of objects using modern methods, natural D*sp*^2^C is presented as an aggregate of nanosized stacks of graphene layers with edge impurities, such as hydrogen, oxygen, nitrogen, and sulfur, which are attached to the carbon core through chemical bonds. Models of the main structural units in the form of framework graphene oxyhydrides of nanometer size are proposed. The composition of the edge oxygen-containing groups allowed separating natural D*sp*^2^C into disordered “C=O” (shungite carbon) and disordered “C=O–C–O” (anthraxolite carbon). This confirms the assumption about the chemical nature of shungite carbon as reduced graphene oxide. The radical nature of the basic structural units makes natural carbon a source of stable radicals.

Shungite carbon is also characterized by curved layers of different lengths covering the pores. A similar bowl-like model of the structure with carbon “cups” (as segments based on which fullerene-like structures are further formed) about 1 nm in size is confirmed by the results of solid-state ^13^C and ^1^H nuclear magnetic resonance spectroscopy [59].

### 3.2. Super-Molecular Structure

**Figure 7:**
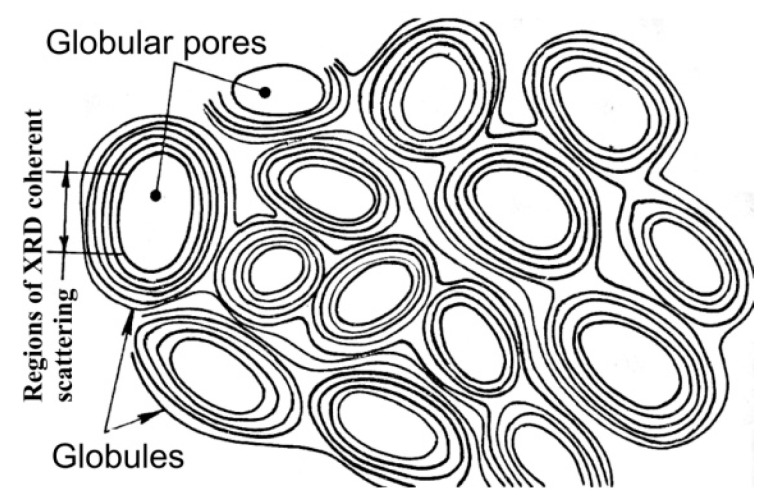
Physical model of shungite carbon. The figure is taken from [61].

For a long time, the super-molecular structure of natural D*sp*^2^C was not the focus of researchers. The interest of physicists and materials scientists in the super-molecular structure of D*sp*^2^C drastically increased after the publication of fullerenes of geological origin found in Karelian shungites [18]. Earlier, Kovalevsky created a model where the fundamental element of shungite carbon is a fullerene-like globule. After publication [18], the Kovalevsky model for a long time became the dominant idea of the super-molecular structure of shungite carbon [60,61]. According to the model, a shungite globule is a spherical or ellipsoidal multilayer carbon formation about 10 nm in size, presumably with a pore inside (Figure 7). The main arguments in favor of the model are the presence of two types of pores (open and closed) in shungite carbon (see [44] and references therein), and the results of processing HRTEM images using the Cowley method [42].

A direct image of the globular structure of shungites was first obtained by scanning tunneling microscopy in [49]. Later, the globular structure of shungite carbon for all major deposits of Karelia and anthraxolites was studied in detail by scanning tunneling and atomic force microscopy [50,51]. Atomic force microscopy visualizes globules of a spherical or ellipsoidal shape, predominantly 20–70 nm in size. There is no regularity in the arrangement of globules; however, regularities of globule aggregation are distinguished [54] due to their grouping into chains (Maksovo, Perya). Similar structures are also characteristic of synthetic glassy carbon [62].

Ribbon-like structures are present in all studied shungites, as well as in most anthraxolites (Figure 3). Such a structure was found only at the beginning of its study using high-resolution transmission microscopy. It is difficult to trace the length and bends of the ribbons from 2D images, however, it can be said that the ribbons have a length of tens and (possibly) hundreds of nanometers and are tangled into rather complex configurations, and at the same time loose knots. Single-layer fullerene-like particles 1–2 nm in size are also often present in the structure of D*sp*^2^C (Figure 3).

A reliable statistical analysis of the ratio of different structural forms of carbon is difficult due to the locality and selectivity of the high-resolution transmission microscopy method. Here, very limited areas are studied, not all structural fragments in which are in a reflective position, and therefore are not visible in the images. The authors of [48] attribute the curvature of primary RGO fragments to the presence of various mineral inclusions accompanying the formation of shungite, from micro–nano-sized particles of silica to nanoparticles of native metals in close proximity to them or in contact with them.

Thus, the complexity of the structure of disordered carbon does not at present allow building a universal model of the structure. This led to the selection of basic elements that formed the structure and determined the most significant properties. This approach was successfully used by Kovalevsky to describe shungite carbon as natural fullerene-like carbon. The globular structural component was singled out by him as the most significant. In [46,47], a flat graphene sheet about 1 nm in size, which is graphene oxide reduced in an aqueous medium, was proposed as the main element. These sheets form stacks, which are further folded into nanosized globules and larger particles. An analogy of the structure of natural D*sp*^2^C is possible within the framework of the ribbon glassy carbon model.

In general, the molecular and super-molecular levels of natural D*sp*^2^C are characterized by mixed structural forms (ribbons and turbostratic packages of graphene layers, multilayer rounded, and flattened globules) based on the graphene grids, which are partially present both in natural (kerogen, anthracite) and technical (glassy carbon, soot) materials. There is a wide range of sizes of structural elements, their shape, and types of defects. These features can be explained by the different origin of the hydrocarbon precursor, the duration and intensity of heating, the magnitude of the applied pressure, as well as a change in the mobility of heteroatoms during the formation process and their initial composition [11].

## 4. Conductive Properties of Natural Disordered *sp*^2^ Carbon

The section will show the static and dynamic conductivity of natural D*sp*^2^C, their dependence on temperature, and current frequency (for dynamic conductivity).

### 4.1. DC Conductivity

Electrical conductivity is one of the key diagnostic features of highly transformed disordered natural carbon, which clearly distinguishes it from all less transformed solid natural hydrocarbon compounds. As a rule, the electrical conductivity of natural D*sp*^2^C is isotropic, and only some samples have a pronounced anisotropy of electrical properties.

The conductivity of D*sp*^2^C is determined by the following factors: (i) concentration of free charge carriers, which is determined by the structure of carbon, the size of the stacks of graphene layers and the distance between them, and their defectiveness, (ii) humidity (ion concentration) over the surface and in the volume of carbon, and (iii) temperature of heating. Here, we will consider the conductivity of dried carbon samples. The largest number of studies of electrically conductive properties at present has been carried out for the carbon of shungites.

**Figure 8:**
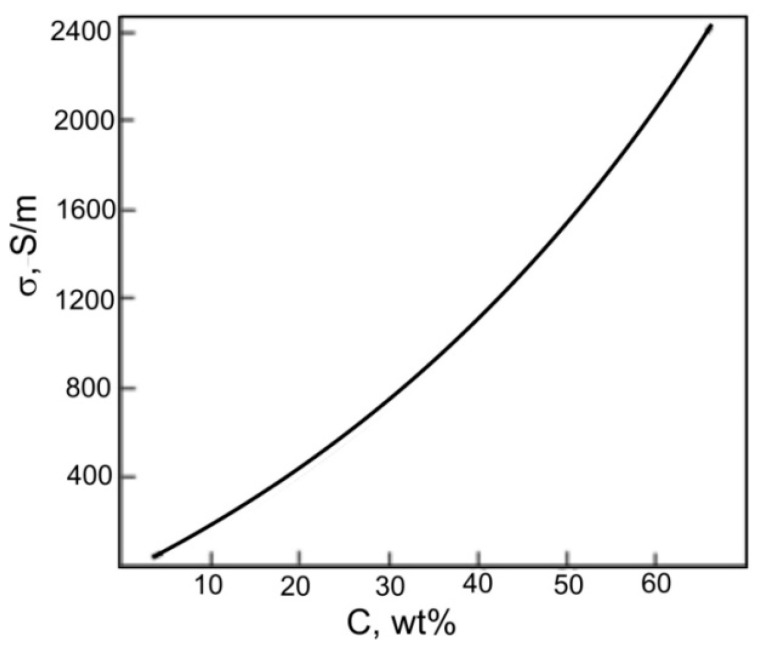
The dependence of the conductivity of shungites on the carbon content according to [64].

Mineral inclusions often occur in carbonaceous rocks. These minerals are generally dielectric and significantly affect the conductivity of the samples. In [63], when studying the electrically conductive properties of 200 samples of shungite in the range of carbon contents from 5 to 65 wt.%, the dependence of the conductivity, *σ,* on the carbon content was revealed, empirically described by the expression:

*σ* = 1.5 *C*^1.87^ (1)

where *C* is the percentage (weight) of carbon content (Figure 8). The electrical conductivity of pure carbon samples from different occurrences is different (Table 1). Considering that in these samples the carbon content is not 100%, but is approximately 90–95 wt.%, then according to Expression (1), the maximum conductivity of the pure carbon phase in shungites was estimated as 8250 S/m. In later studies [64], the conductivity of shungite from Shunga was determined as 8000 S/m, which is generally comparable with the results [63]. Thus, the conductivity of shungites is comparable to the conductivity of natural graphite across the graphene layers [65].

Temperature dependences for resistivity (*ρ*), Hall mobility (*μ*), and Hall coefficient (*RH*) in the temperature range T = 80–500 *K* in shungite were described in [66]. At room temperature, shungite carbon showed the following parameters: *ρ* = 3.53 · 10^−3^*Ω* cm, *μ* = 8.0 cm^2^/*V* s, *RH* = −2.83 · 10^−2^ cm^3^ C^−1^, and charge carrier concentration *n* = 2.2 · 10^20^ cm^−3^. At the same time, *ρ*, *μ,* and *RH* weakly depend on the change in temperature. The author draws attention to the similarity in the behavior of the temperature dependence of the Hall coefficient in shungite carbon and in single-crystal graphite. In both cases, *RH* has a negative sign and a small absolute value. Additionally, the Hall coefficient for shungite is comparable to the Hall coefficient for low-temperature (temperature 1300–1600 °C) glassy carbon, which is also temperature-insensitive [67]. It is interesting that the Hall coefficient for high-temperature glassy carbon (above 1700 °C) already has a positive sign. At the same time, the dependences of ρ and μ from temperature differ qualitatively from those for single-crystal graphite.

**Figure 9:**
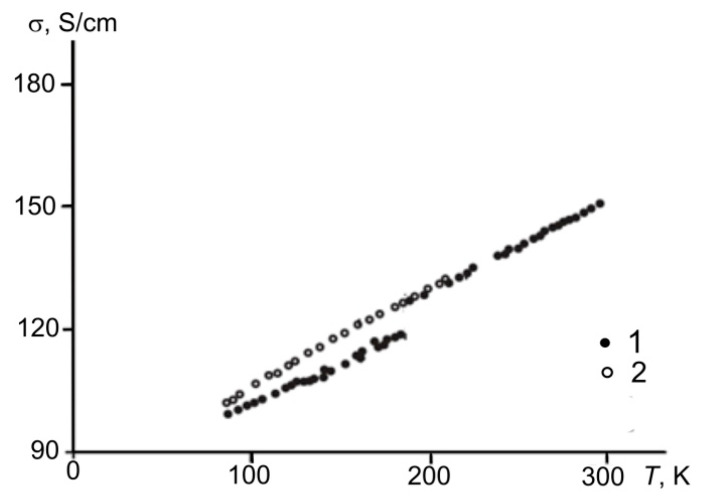
Temperature dependence of the electrical conductivity of shungite carbon upon cooling from 300 K (1) and heating from 77 K (2) (adapted from [68]).

The direct current conductivity of shungite carbon from the Shunga and Maksovo occurrences at low temperatures was estimated in [64,68], and an example of dependence, *σ*(*T*), is shown in Figure 9. This study showed a higher conductivity of shungite from Maksovo. The conductivity of shungite from Shunga slightly depends on the temperature. The dependence of the electrical resistance of Maksovo and Shunga shungites on temperature upon heating [69] shows a decrease in resistance typical of the semiconductor type of conductivity upon heating from room temperature to 900 °C.

**Table 1:** Conductivity of some natural D*sp*^2^C disordered carbon samples.

Sample, Occurrences	Conductivity (S/m)
Shungite (ShSh1), Shunga, Karelia	8000 [64]; 4700 [63]; 4670 [69]; 14,600 [68]
Shungite (ShM1), Maxovo, Karelia	2000 [63]; 1640 [69]; 1200–1500 [our data]
Shungite (ShCh1), Chebolaksha, Karelia	1100–1200 [our data]
Shungite (ShN1), Nigozero, Karelia	1250 [63]
Anthraxolite (ANZPr), Perya, Novaya Zemlya Island, Russia	200–600 [our data]
Anthraxolite (Columb), Colombia	0.5–1 [our data]
Anthraxolite (ANZPa), Pavlovo, Novaya Zemlya Island, Russia	100–300 [our data]
Anthraxolite (ABak), Bakyrchik, Kazakhstan	0.1–0.3 [our data]

Similar results to the semiconducting type of dynamic conductivity of the shungite carbon from the Shunga and Maksovo occurrences was found in [70]. The behavior of the electrical conductivity in the temperature range of 77–300 K was similar to that described for semiconductors. At low temperatures, there are three successive types of dependence. First, growth occurs at the donor type of conductivity. At temperatures below 1/2Θ (where Θ is the Debye temperature), electrons scatter on impurities or lattice vibrations, so the electrical conductivity decreases with increasing temperature. At temperatures above 1/2Θ, the electrical conductivity starts hopping and increases again with increasing temperature [71].

In general, the nature of the electrical conductivity of natural D*sp*^2^C at direct current is ambiguous. Here, perhaps, we meet the influence of the geological origin, where there are some differences in the structure and elemental composition of the samples even within the same occurrence. From this point of view, studies of electron paramagnetic resonance (EPR) spectra are interesting [11]. The EPR spectrum of natural carbon compounds can be conditioned by both conduction electrons (an example of graphite is given in the work [72]) and unpaired electrons in free radicals (charcoal, anthracite [73]) or transition metal complexes. The works [64,74] indicate that the EPR signal of shungite is mainly conditioned by conduction electrons with Pauli-type magnetism. The authors give the calculated number of conduction electrons as 1.33 · 10^19^ spin/g. The same order of magnitude of the number of unpaired electrons (1–8 · 10^19^ spin/g) is given in [75] for shungites from Shunga, Maksovo, Nigozero, and Chebolaksha occurrences. Thus, the EPR signal can be divided into two components: the first is associated with localized unpaired electrons (or with electrons in which delocalization is limited), and the second, independent of temperature, is conditioned by conduction electrons [11]. On the other hand, a dependence of the conductivity of shungite during high-temperature heating, similar to polycrystalline disordered graphite and synthetic glassy carbon, indicates the proximity of the semiconductor’s type of conductivity.

The conductivity of anthraxolites at direct current is much worse than that of shungite carbon from Karelia. At best, the conductivity of anthraxolites is hundreds of Sm/m (Table 1). For example, the conductivity of anthraxolites from various occurrences of Novaya Zemlya Island, Russia, varies from 200 (Pavlovo occurrence) to 600 S/m (Perya occurrence), and the conductivity of anthraxolites from Kazakhstan, the Crimea, and Colombia is tens to fractions of S/m.

**Figure 10:**
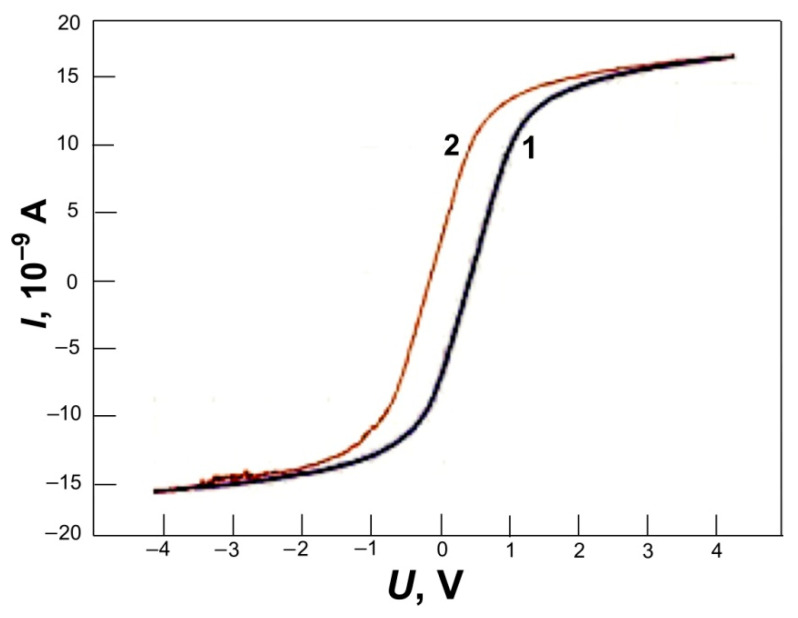
Hysteresis in the *I*-*V* curve of shungite (electric force spectroscopy mode of AFM). The curves were measured at one point with an increase in the voltage (curve 1) and with a decrease in the voltage (curve 2).

Interesting local features of conductivity were found using atomic force microscopy [76,77]. Local areas on the carbon surface of shungites with dielectric properties were found using spreading resistance microscopy and electric force spectroscopy [77]. In the region of both positive and negative applied voltages, shungites and anthraxolites have hysteresis of volt-ampere characteristics (VAC) in separate areas (Figure 10). For the previously described nanogranular metal-dielectric composites, such a hysteresis of VAC is explained by the processes of polarization of the dielectric component [77]. It can be assumed that at the points of taking such VACs on the surface of shungite, the amount of impurity elements (chlorine, nitrogen, oxygen, sulfur) locally reached values significant for changing the electrically conductive properties of the surface.

### 4.2. AC Conductivity

**Figure 11:**
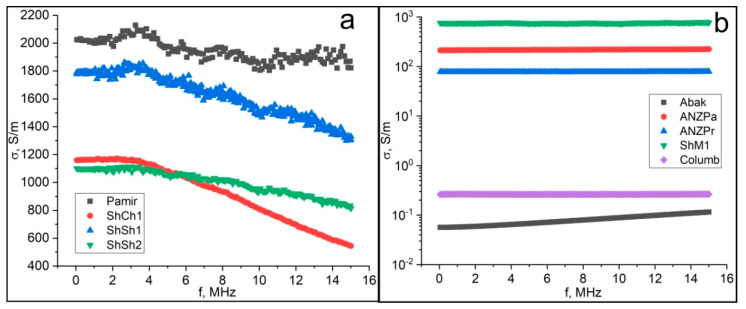
Frequency dependence of the conductivity of the first type (**a**) and the second and third types (**b**). Samples code: Pamir—natural graphite; ShCh1, ShSh1, ShSh2, ShM1—samples of shungite carbon from various occurrences of Karelia; anthraxolites from Kazakhstan (Abak), Novaya Zemlya islands (ANZPa, ANZPr), and Colombia (Columb).

Let us consider the frequency dependence of the electrical conductivity of natural D*sp*^2^C. We have found three fundamentally different types of such a dependence, which allows dividing the samples of D*sp*^2^C into three groups (Figure 11). The first group of samples shows a complete absence or a weak dependence in the low-frequency region up to a frequency of 2–4 MHz. With a further increase in frequency to 15 MHz, the conductivity begins to decrease sharply, by one and a half to two times. These samples include a reference sample of natural graphite and three samples of Karelian shungite. Samples of anthraxolites, one of the samples of Karelian shungite, and a sample of Kozhim carbon show an almost complete absence of dependence of conductivity on the frequency of alternating current up to a frequency of 15 MHz. Finally, the Kazakhstan anthraxolite sample shows a continuous linear weak increase in conductivity with increasing frequency.

**Figure 12:**
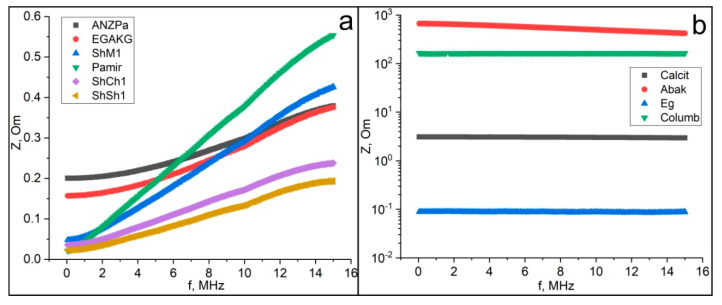
The dependence of the impedance on the frequency (**a**—samples with an increase in impedance. **b**—samples with a constant or decreasing impedance). Samples code: Pamir—graphite; ShCh1, ShSh1, ShSh2, ShM1—samples of shungite carbon from various occurrences of Karelia; anthraxolites from Kazakhstan (Abak), Novaya Zemlya islands (ANZPa, Calcit, Eg), Colombia (Columb), and Subpolar Ural, Kozhim river (EGAKG).

The frequency dependence of the total electrical resistance (impedance) for our samples divides them into two groups (Figure 12). In the first group, the impedance increases linearly and monotonically with increasing frequency. This group includes samples of Maksovo, Shunga, and Chebolaksha shungites, Pamir graphite, anthraxolites from Novaya Zemlya islands, and disordered carbon from Subpolar Ural (Kozhim), Russia. In the second group, represented by the remaining anthraxolites, the impedance slightly decreases with increasing frequency.

The presence of such a dependence is determined by the reactive component of the resistance (capacitive or inductive). According to the measurement results, the dependences of the inductance on frequency were found and evaluated in all samples of shungite and in most samples of anthraxolites, with the exception of Kazakhstan and Colombian anthraxolites, in which a capacitive dependence was found.

It can be assumed that the super-molecular structure is influencing here. The type of resistance (inductive or capacitive) is formed by the elements prevailing in the super-molecular structure, which primarily include stacks of graphene layers and ribbons. The capacitive type of resistance of the Bakyrchik and Colombia samples can be associated with their dominant stacks structure.

**Figure 13:**
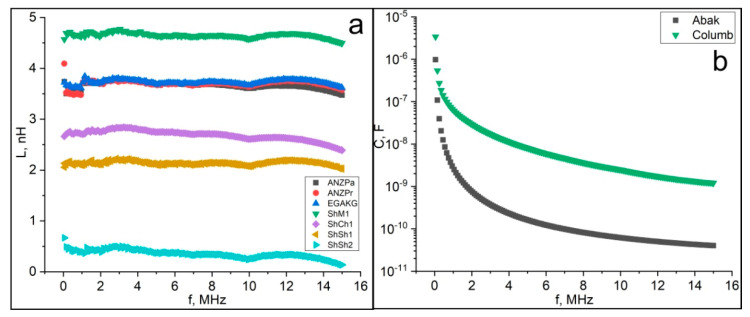
Frequency dependence of inductance (**a**) and capacitance (**b**). Sample code is similar to Figure 12.

For the vast majority of samples, the reactance increases almost linearly with frequency. In the case of an inductive type of resistance (reactance X=ωL, where ω=2πf) with increasing frequency, *f*, the reactance, *X,* also grows linearly, so the inductive resistance, *L,* practically does not depend on frequency and does not contribute to this dependence (Figure 12 and Figure 13). In the case of a capacitive type of resistance (reactance $X=\frac{1}{\omega C}$) for the Abak and Columb samples, it is the capacitance that makes the determining contribution (see Figure 13). In each frequency interval, the capacitance decreases more than the frequency increases, which determines some increase in reactance.

**Figure 14:**
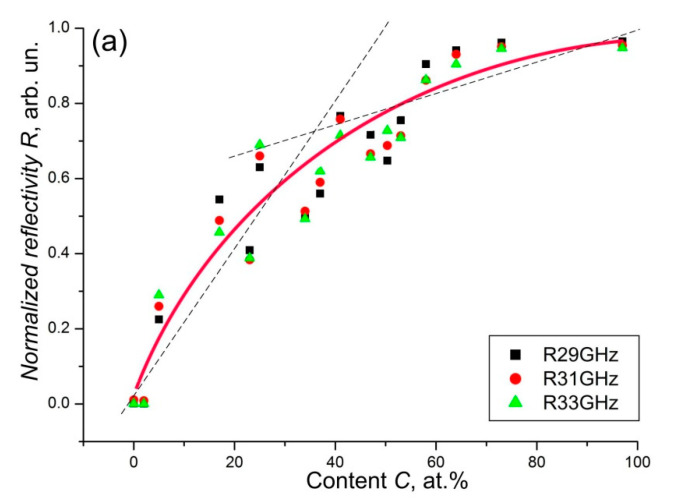

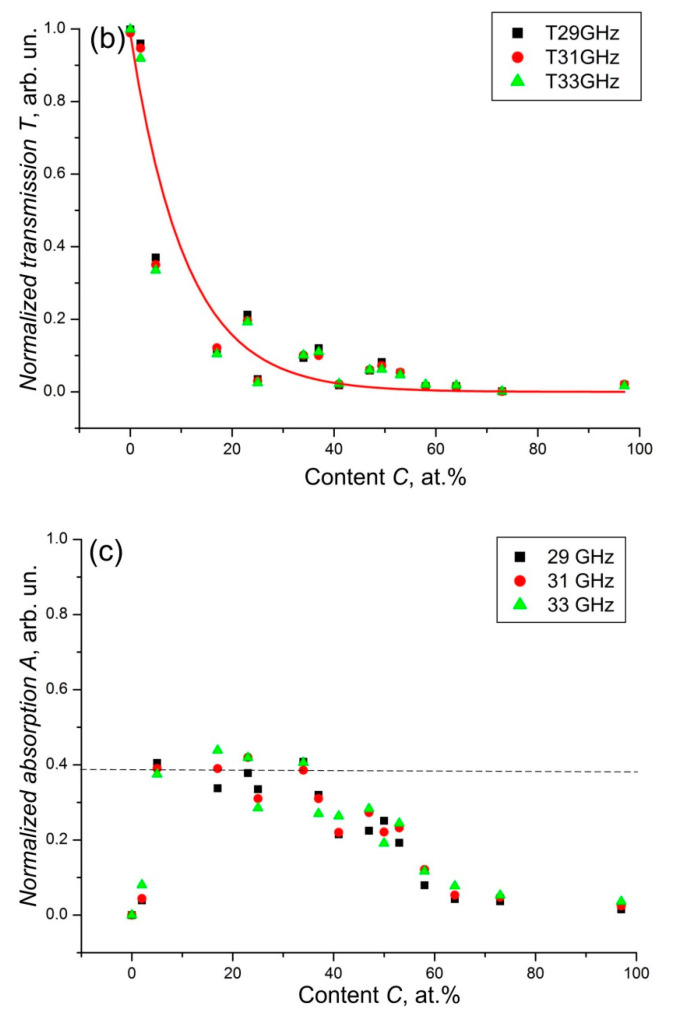
The dependence of the reflection (**a**), transmission (**b**), and absorption (**c**) of electromagnetic radiation on the carbon concentration of shungite plates in the frequency range 26–38 GHz. Approximation (red line in (**a**,**b**)) is performed at a frequency of 31 GHz (from [80]).

As our studies have shown, samples from the Maksovo and Shunga deposits exhibit high microwave reflective and conductive properties [78,79]. Even ultrathin shungite plates with a thickness of 8–15 µm in the ranges of 8–12 and 26–38 GHz reflect (95 ± 2)% of the total incident radiation, and the shielding efficiency reaches almost 100% (Figure 14) [80]. It should be noted that the microwave reflective properties of shungite plates are practically independent of frequency, keeping them in a wide range [79]. Surprisingly, shungites have a high reflection of microwave radiation even with not too high static conductivity. Metals achieve this efficiency of microwave reflection at conductivity higher by one or two orders of magnitude.

Theoretical evaluation of the dynamic conductivity of Maksovo and Shunga shungite samples at gigahertz frequencies by measuring the microwave reflection from ultrathin (8–15 µm in thickness) shungite plates showed that the dynamic conductivity in these frequency ranges was about one and a half times higher than the conductivity at direct current [79]. Thus, the frequency dependence of the conductivity of natural disordered carbon in a wide (kilo-, mega-, and giga-hertz) frequency range is ambiguous. In the megahertz range, the conductivity decreases with frequency; in the gigahertz range, the dynamic conductivity exceeds the static one.

## 5. Models of Electrical Conductivity

The conductivity features of natural D*sp*^2^C are determined by its multilevel structure. At the molecular level, these are graphene layers, partially curved and with defects; at the supramolecular level, these are nanosized packs of graphene layers, multilayer ribbons, and globules that form larger aggregates (blocks, chains), reaching micrometer sizes. Additionally, microporosity affects on the electrical conductivity. Let us consider these structural features sequentially.

### 5.1. Molecular Conductivity

**Figure 15:**
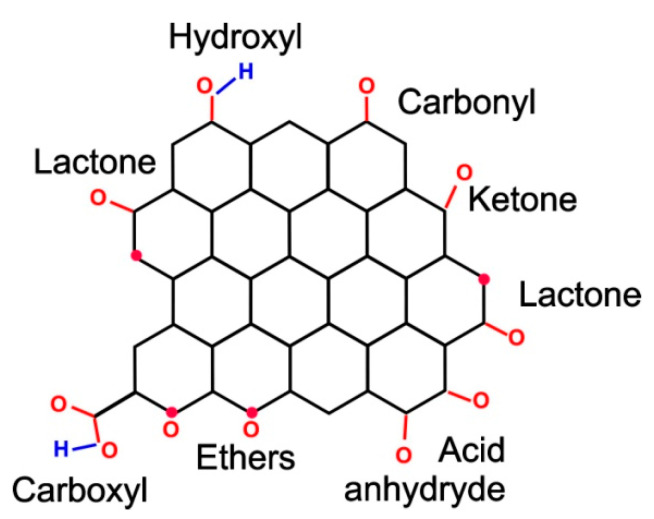
Typical oxygen-containing functional groups framing a graphene layer in natural D*sp*^2^C.

At the molecular level, in disordered graphitic carbons, electrical conductivity generally increases with an increase in the ratio of *sp*^2^/*sp*^3^-hybridized bonds due to the delocalized electrons introduced by the *sp*^2^ bond [81]. This delocalization facilitates the transport of electrons in the plane of the graphene layer, making it highly conductive, in contrast to the high resistivity in systems containing a rigid network of localized *sp*^3^ bonds [82]. Additionally, edge functional groups, such as carbonyl, carboxyl, and lactone, which frame the edges of graphene layers in disordered carbon (Figure 15), significantly affect molecular conductivity [6,52]. The presence of such functional groups and the fragmentation of the structure contribute to a significant decrease in electrical conductivity. This relates to the destruction of the *π*-conjugated system of bonds and their overlapping by the indicated groups. The removal of most functional groups from the edges of graphene layers during the thermal and chemical transformation of bitumen in the geological environment results in the rearrangement of the π-conjugated system. The increase in conductivity occurs due to the removal of the functional groups of the base planes in the packs of graphene layers in the process of reduction of mobile oxygen-containing groups and destruction of hydrogen-containing groups. The key importance of such a chemical transformation with the removal of functional groups is shown in [83], where the thermal destruction of hydrogen-containing groups in bitumen of an average degree of transformation contributes to the appearance of electrical conductivity before the start of graphite-like ordering of the structure. The last factor distinguishes the molecular mechanisms of the conductivity of synthetic glassy carbon and natural disordered carbon, since glassy carbon is much more “pure” and functional groups practically do not affect its conductivity.

Thus, in disordered carbon, one can note the key importance of intrinsic electronic conductivity, which is conditioned by the presence of a large number of honeycomb graphene structures. It is likely that an additional contribution to the conductivity can be made by impurity *p*-type conductivity associated with the presence of heteroatoms at the edges of the graphene layers. These chemical elements (primarily oxygen) play the role of donors, and when interacting with the molecular structure, they create defects, which contributes to an increase in the number of free charge carriers (electrons). However, taking into account the low concentrations of heteroatoms remaining in the structure of the considered carbon materials, the contribution of the *p*-type conductivity can be considered significantly less significant than that of the *n*-type conductivity [11].

EPR studies also present a large number of conduction electrons with Pauli-type magnetism [64,74]. In addition, shungites and anthraxolites contain localized unpaired electrons not associated with π-orbital states, which can be included in chain carbon compounds at the edges of graphene layers, and free bonds of σ-orbitals with *sp*^2^ carbon atoms in the composition of functional groups [11]. Part of the sigma-free bonds can be neutralized by heteroatoms. The unsaturation of these bonds is probably conditioned by the complex structure of disordered carbon, since they can be located in the gaps between the graphene layers or in closed pores, where the access of non-carbon atoms is difficult. It is noteworthy that the shape of the EPR signal and its temperature dependence for shungites and anthraxolites are similar to the EPR characteristics of onion structures and other materials where carbon is in different hybrid states.

### 5.2. Super-Molecular Conductivity

The fundamentally different nature of the frequency dependence of conductivity and different types of resistance of shungite and anthraxolite samples from different deposits indicate that the super-molecular structure of natural D*sp*^2^C is very diverse. Even samples with similar molecular structures can show different types of conductivity, which indicates the predominant influence of nanosized structural elements. As mentioned above, the main structural units in our samples are bundles of often distorted and curved nanoscale graphene layers, multilayer ribbons, and (to a lesser extent) closed multilayer globules.

**Figure 16:**
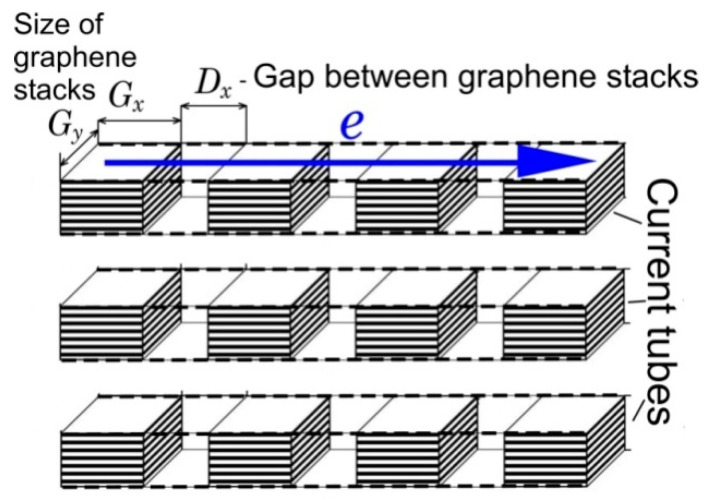
Model of conduction of disordered carbon within current tubes of sequentially arranged stacks of graphene layers.

An essential role in conductivity is played by the sizes of super-molecular structural elements. If the sizes of stacks of graphene layers < 100 nm, then the mechanism of scattering of charge carriers at inter-pack boundaries is dominant [84]. This scattering is largely determined by the distance between the stacks. A theoretical analysis of the effect of the nanostructure on the conductivity of D*sp*^2^C based on the structure as a set of stacks of graphene layers was carried out in [85,86,87]. Here, the chaotic nature of the structure of shungite carbon was reduced to a regular model of parallel conductive tubes from sequentially arranged stacks of graphene layers with dimensions of a few nanometers (Figure 16) [86]. The comparison of the model with the real structure was carried out on the example of two shungite samples from the Maksovo and Nigozero occurrences, in which the average sizes of the stacks and the gaps between them were estimated using high-resolution transmission microscopy methods [85,87]. In these samples, the average sizes of packs of graphene layers and the gaps between them differ significantly. The study of the dependence of the resistance of the gap between the stacks on the size of the stacks and the size of the gap between them showed that the resistance of the gap significantly decreases with the increasing size of the stacks, but at the same time reacts poorly to an increase in the gap size, and the resistance of the gap between the stacks is inversely proportional to its size. The model of current tubes allows establishing a correspondence between conductivity and sizes of gaps and stacks of graphene layers. These calculations showed the key effect of gap size and orientation of graphene stacks on conductivity versus stack size [85,87]. This model is the first approximation to the electrically conductive model of shungite carbon, and on its basis a general conductivity model for D*sp*^2^C can be developed.

Nevertheless, in a constant electric field, electrons pass through the sample throughout its entire volume: through the boundaries of blocks and globules, along graphene layers, and from layer to layer. Some of the conductive bonds are likely to have high-energy barriers, so there may be several key paths with the least resistance for current to flow. Indeed, even at a low temperature, effective conductive bonds are preserved. According to the authors of [64], this feature distinguishes natural D*sp*^2^C, for example, from artificial activated carbon fibers, in which with decreasing temperature, the resistance increases ten-fold. This indicates the high quality of electrical contacts between the super-molecular structural units of D*sp*^2^C.

In some samples, a significant increase in reactance with frequency indicates an inductive type of conduction. Previously, non-conductive inclusions up to several micrometers in size were found in shungite samples, which are covered with a thin film of well-ordered graphitic carbon. Such carbon structures can act as inductors [88,89]. As well as inductors, multilayer bent ribbon structures of carbon can act, which are randomly intertwined and have a length of up to hundreds of nanometers and units of micrometers.

The high conductivity of nanoscale structural elements of shungites is evidenced by their high shielding properties. In the most general case, the shielding properties of shungite are determined by its conductivity. The chaotic structure of the conducting regions ensures the isotropic nature of the conduction. Dynamic microwave conductivity is provided by currents circulating inside the local conductive sections. That is, the dynamic conductivity can be quite high even in the absence of direct contact on direct current or with a significant resistance of the gap between the carbon structural elements.

**Figure 17:**
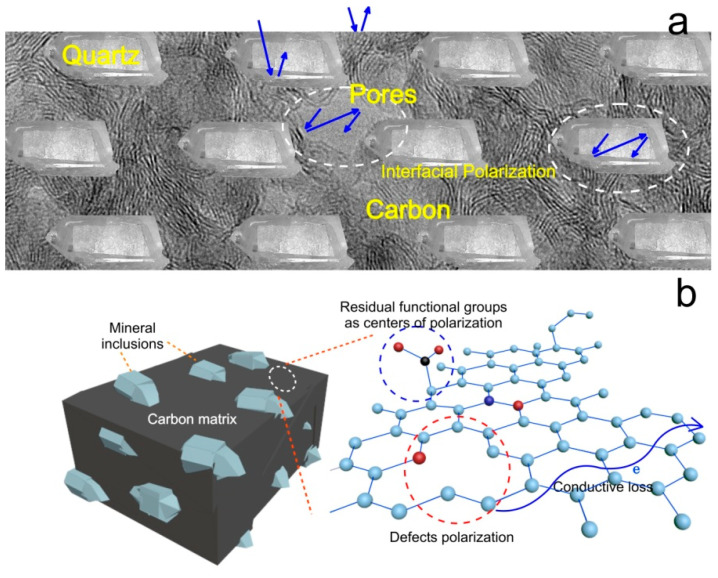
Main mechanisms of shielding electromagnetic radiation in a mixed carbon–mineral composite ((**a**), from [81]) and shungite carbon (**b**). Blue arrows show direction of microwave radiation.

In [80,90,91], schemes were proposed for the interaction of electromagnetic radiation with shungite carbon (Figure 17). Although, it should be noted that in [90,91], the pressed powder from the shungites of the Zazhoginsky occurrence with a carbon content of about 30% was studied. It is shown that the shape of the transfer coefficient curve for shungite powder is typical for broadband absorbers, which may be due to high dielectric losses. The authors of the papers do not rule out that multiple re-reflections and absorptions take place in layered carbon structures. This is accompanied by a decrease in the resulting electrical component in the direction of transmission. At the same time, multiple internal reflection of electromagnetic waves is provided precisely by carbon micro- and nano-structures and porosity. In addition, the absorption properties are improved even with a small content of heteroatoms (H, O, N, S), which produce polarization centers and enhance absorption properties [80].

Thus, the most significant features that determine the electrically conductive properties of shungite are conditioned by: (i) nanosized graphene layers, (ii) bending of graphene layers, indicating the presence of topological defects and the appearance of *sp*^2^ hybridization, and (iii) a significant amount of edge states at the boundaries of open fragments of graphene layers terminated by functional groups. With a high probability, there is a double mechanism of electron transport in natural D*sp*^2^C. The first is conditioned by the presence of stacks of graphene layers and multilayer ribbons, within which the ballistic path predominates, as in ordered graphene. Between the stacks of graphene layers, electrons are scattered by defects and impurities; therefore, the mechanisms of electron tunneling through potential barriers and diffuse mechanisms are actively switched on here.

## 6. Technological Applications of Electrophysical Properties of Natural Disordered Carbon

Recently, many studies have been carried out on various properties of natural D*sp*^2^C in technological applications, and electrical conductivity is among the most demanded properties.

One of the most promising directions for the use of natural D*sp*^2^C is the use of composite radio shielding materials as an active filler. The impact of electromagnetic radiation of various nature on technical and biological objects significantly affects their functioning. A significant increase in the number of sources of electromagnetic radiation (mobile radio communications, computer equipment, overhead power lines, radar stations) makes protection against electromagnetic effects in a wide frequency band very relevant [92,93,94,95,96,97]. At present, biomedical, hygienic, and environmental aspects of electromagnetic radiation have acquired particular relevance. The most effective from the point of view of shielding electromagnetic radiation metal screens, which have high conductivity and, accordingly, strong reflection, interfere with the radio-electronic equipment operating in the room and create an unfavorable electromagnetic environment. Therefore, various loss mechanisms must be implemented in the screens: dielectric and magnetic losses, dispersion, diffraction, interference, and internal re-reflections of electromagnetic waves, causing additional weakening of the energy of an electromagnetic wave due to Rayleigh scattering, addition of waves in antiphase, etc. Shungite powder, due to its inhibitory properties, composite composition, low cost, and lightweight can be used as a component for the production of materials that provide effective shielding of electromagnetic radiation [70,90,98,99,100]. In addition, for example, shungite materials at a certain concentration of carbon have not only reflective, but also good absorbing properties, which expands the possibilities of their use for shielding electromagnetic radiation. For example, recently, from shungite, flexible ultrathin (about 10 μm in thickness) plates were produced with shielding of almost 100% of the incident microwave radiation in the frequency ranges of 8–12 and 24–36 GHz, while absorbing half or most of the radiation [80]. The results of studies of the effect of conductive carbon filler have been published in many works, for example [92,93,94,95,96,97,101]. Shielding materials based on polymers with similar protection characteristics are several millimeters thick. Therefore, materials based on natural carbon with a thickness of about 10 μm can be used in technological devices in conditions of very limited space. The electromagnetic radiation protection characteristics of carbon-based geomaterials can be easily adjusted and improved by adjusting the appropriate carbon content of the material or by modifying them thermally or chemically [70,102,103,104,105].

The electrically conductive properties of D*sp*^2^C can be used in construction and transport. Cement-based electrically conductive composites are a typical example of multifunctional composite materials. For electrical conductivity in the cement matrix, it is necessary to introduce electrically conductive additives, among which carbon fillers are widely used, including materials based on graphite [106]. Changes in temperature or mechanical stress change the electrical properties of such cementitious composites, allowing them to be used, among other things, as temperature or voltage sensors for detecting damage in building structures, weighing vehicles in traffic, or monitoring the temperature of pavement or building walls. In [107], it is shown that shungite carbon is a very promising electrically conductive additive for cement composites. The introduction of at least 16 vol.% shungite imparts electrically conductive properties to cement composites and allows them to be used for stress/damage or temperature monitoring with high sensitivity. Radio shielding of walls is also of interest [108]. A thermoelectric effect has been found in cement composites with shungite carbon. Similar properties are expected for materials filled with disordered carbon from the Bakyrchik occurrence, East Kazakhstan [109,110].

Carbon materials are promising components for energy storage devices, such as supercapacitors and lithium-ion batteries, due to the reversible reaction between carbon and lithium-carbon compounds [111,112,113]. Intensive research is now underway on various carbon structures, aimed at finding the most effective and affordable materials for achieving high storage capacity of lithium ions in the field of renewable energy sources. Among these structures, much attention is paid to multilayer graphene [114,115,116]. To achieve a high storage capacity of lithium ions in synthetic carbon materials, functional groups, surface area, pore size distribution, and other parameters are widely varied. All these characteristics vary widely in natural D*sp*^2^C from different occurrences. The research results published in [117] showed that shungite carbon can serve as an alternative and effective resource for lithium-ion battery electrodes. Already after simple processing, the storage capacity of shungite material (approximately 400 mAh/g) significantly exceeds the capacity of graphite, which also has a limitation (372 mAh/g). This ability is probably related to the disordered structure of the stacks of graphene layers and their partially open edge atoms. Further research in this area can contribute to the production of environmentally stable and cheap materials with high electrochemical characteristics based on disordered natural carbon.

Advances in the production of polymers with intrinsic electrical conductivity have not canceled the production of polymers filled with an electrically conductive filler, due to the rather complicated synthesis and instability of conductive polymers, as well as the difficulty in obtaining samples that combine good electrical and physical-mechanical characteristics. Such properties of natural D*sp*^2^C as good compatibility with both polar and nonpolar polymers turned out to be important to create polymer composite materials with electrically conductive properties, which makes it possible to obtain compositions with high degrees of filling. In turn, this allows varying the conductive properties of polymers over a wide range, and to obtain polymers with more stable characteristics, including when exposed to different temperatures [118].

A complete and detailed review of the areas of technological application of synthetic D*sp*^2^C (glassy carbon) was presented in [1,80]. The similarity of the structural characteristics and electrophysical properties of natural and synthetic D*sp*^2^C suggests the interchangeability of these materials in technological processes. For example, the use of glassy carbon as an antistatic agent in electrically insulating thermoplastic polymers has recently been proposed for the production of antistatic packaging, which is used in the electronics industry to protect electronic components from electrostatic discharge. Glassy carbon particles form electrically conductive paths in such polymers and avoid the accumulation of electrostatic charges [119]. As shown above, natural D*sp*^2^C can also be successfully used to produce conductive polymers. However, some chemical differences caused by differences in precursor hydrocarbons, and some instability in the composition and properties of natural material, do not allow replacing one material with another without additional studies.

## 7. Conclusions

Natural disordered *sp*^2^ carbon has a diverse molecular and chemical structure, which determines the diversity of its electrophysical properties. Natural D*sp*^2^C is almost a complete analogue of glassy carbon in terms of properties and structure and can be considered as an environmentally friendly and cost-effective replacement for glassy carbon in these applications. The diversity of the molecular and super-molecular structure and the features of the chemical composition of impurities, despite their relatively low content (a few percent), determine the variety of electrical properties. The wide range of absolute conductivity, such as resistance (inductive and capacitive), and the presence of various effects (Hall and others), makes natural disordered carbon a promising object for various technological applications. At present, D*sp*^2^C has a good prospect for use in energy storage for the manufacture of materials that shield and absorb high-frequency electromagnetic radiation.

Natural materials are interesting because the synthesis of such materials is difficult. In geological conditions, the processes of their formation take hundreds of thousands and millions of years, and almost all chemical reactions are completed. There is no such amount of time in the laboratory so it is necessary to use catalysts to speed up chemical reactions. This leads to possible changes in the structure of the material and additional costs for the search of catalysts. Natural material, despite its frequent variability in composition and structure, has some unique properties that are difficult or impossible to obtain in the laboratory, so it is important to keep such materials in the focus of attention of researchers in various fields of science.

## Data Availability

Data sharing is not applicable for this review.
